# Special Issue “Drugs in Inflammatory Bowel Diseases”

**DOI:** 10.3390/ijms262311611

**Published:** 2025-11-30

**Authors:** Vanessa D’Antongiovanni, Nunzia Bernardini, Carolina Pellegrini

**Affiliations:** Unit of Human Histology and Embryology, Department of Clinical and Experimental Medicine, University of Pisa, 56126 Pisa, Italy; nunzia.bernardini@unipi.it (N.B.); carolina.pellegrini@unipi.it (C.P.)

Inflammatory bowel diseases (IBDs) comprise a group of idiopathic, chronic, relapsing, and inflammatory conditions, which include ulcerative colitis (UC) and Crohn’s disease (CD) [1]. These disorders are characterized by intestinal symptoms, such as gut dysmotility and visceral pain, associated with chronic inflammation of the intestinal mucosa [1,2]. A common feature among IBD patients is the dysregulation of intestinal mucosa functions along with an exuberant presence of activated immune cell populations [3,4,5]. Indeed, it is widely recognized that in genetically susceptible hosts, pathologic immune responses with an imbalanced interaction with luminal host microbes compromise the intestinal epithelial barrier (IEB), triggering the development of chronic intestinal inflammation [3,6]. In light of this, an interesting review by Zhu et al., which is included in the present Special Issue, describes the impact of the gut microbiota on IEB alterations and enteric inflammation underlying the IBD pathogenesis [7]. In particular, the authors describe how manipulation of the gut microbiota through probiotics or fecal microbiota transplantation (FMT) may improve IBD symptomatology by strengthening the gut barrier, with consequent reductions in intestinal inflammation [7]. Of note, in the same review article, the authors provided an exhaustive overview of the effects of traditional and biotechnological drugs on the host gut microbiota, showing that some drugs, such as mesalazine, which is widely used for UC patients, promote gut microbiome diversity, increasing the proportion of beneficial bacteria at the expense of harmful species [7,8,9]. Overall, these findings highlight the clinical relevance of drug–microbiota interactions in this pathological context, as well as the putative role of gut microbiota manipulation in alleviating IBD symptomatology.

At present, the pharmacological management of IBD patients is far from satisfactory [10,11,12,13]. Indeed, the insufficient efficacy and safety of traditional drugs (i.e., corticosteroids, immunosuppressants, and salicylates), as well as recent biotechnological drugs (including anti-TNF and other biologic treatments), have spurred the interest of the scientific community, who have sought to identify novel molecular targets for the management of the immune inflammatory components and visceral hypersensitivity associated with IBDs. In this setting, long non-coding RNAs (lncRNAs), a class of RNAs with a length of over 200 nucleotides, have emerged as important players in shaping intestinal inflammation and immunity in CD patients. In particular, the lncRNA expression profile in IBD patients seems to be correlated with the response to biologic therapy, including anti-TNF therapy [14,15]. However, several aspects remain to be clarified. In this perspective, the paper by Baldan-Martin analyzed the transcriptomic profile of terminal ileum and left colon biopsies from CD patients (both active and quiescent disease), along with those from healthy controls, demonstrating a different lncRNA expression profile between ileal and colonic tissues [16]. In particular, the expression levels of some lncRNAs were positively associated with inflamed areas of intestinal tissue, thus suggesting a correlation between lncRNAs and disease activity. In addition, the authors performed ex vivo treatment of biopsies with Infliximab; no modulatory effects were observed when comparing lncRNA transcriptome levels against untreated biopsies, which was in accordance with both intestinal location and the presence of inflammation (active vs. quiescent disease) [16]. Of note, although this paper shows several limitations, as reported by the authors, including the limited number of patients and healthy controls, as well as the lack of a potential mechanisms of action of the lncRNAs identified, it contributes to the improvement of treatment for IBD by providing molecular readouts of treatment effects, potentially aiding in the selection of responders and non-responders. In this setting, another therapeutic strategy for the management of IBDs is represented by natural compounds [17,18]. In particular, the review article by Li et al. reported the beneficial effects of pomegranate and its ellagitannins in counteracting chronic intestinal inflammation associated with UC through the inhibition of the NF-κB, MAPK, p70S6K, and STAT3 pathways [19]. In addition, several studies on human intestinal epithelial Caco-2 cells, incubated with lipopolysaccharide, reported the anti-oxidant and strengthening effects of pomegranate and its tannins on IEB [19]. These positive effects of pomegranate and its tannins were corroborated by in vivo studies on mice with dextran sulphate-induced colitis, showing an increase in the expression of tight junction proteins, zonulin-1 and occludin, along with a reduction in colonic MPO levels, which contributed to decreased colonic inflammation and improved IBD symptoms [19].

Of interest, more than 70% of IBD patients experience visceral pain, a pathological condition that arises from increased sensitivity of the internal organs due to persistent inflammation [20,21]. At present, the pharmacological management of visceral pain associated with IBD remains ineffective [22]. In this regard, acetyl-L-carnitine (ALCAR), an acetylated form of the naturally occurring amino acid carnitine, has been shown to exert pain-relieving effects in various chronic pain conditions, with good tolerability [23,24]. On these bases, the interesting study by Lucarini et al. investigated potential anti-nociceptive effects of ALCAR in a model of persistent visceral pain resulting from colitis induced by the intrarectal injection of 2,4-dinitrobenzene sulfonic acid (DNBS) in rats [25]. The authors observed that ALCAR significantly reduced the establishment of visceral hyperalgesia in DNBS-treated animals. In addition, ALCAR treatment partially reduced colon damage in rats, counteracting enteric glia and spinal astrocyte activation resulting from colitis as well as protected enteric neurons from the inflammatory insults [25]. Overall, these findings suggest the putative usefulness of ALCAR as a food supplement, which might be integrated into the therapy of IBDs as an adjuvant strategy to either prevent or counteract visceral pain.

It is well known that the chronicization of the inflammatory process favors the development of microniches that support the development of neoplasia [26,27]. Indeed, IBD patients have a 3–6-fold increased risk of developing colorectal cancer (CRC) compared to the general population [28,29,30]. In this setting, radiotherapy remains one of the most common and effective cancer treatments, although about 20–40% of patients are resistant or minimally responsive to radiotherapy [31,32]. For this reason, radiosensitization is one innovative method used to enhance radiation sensitivity. Abdelaziz et al. investigated the role of cathepsin L, a member of the cysteine cathepsins involved in radiation sensitivity, in combatting colon carcinoma Caco-2 cells [33]. The authors observed that cathepsin L was upregulated in response to radiation treatment, compared with non-irradiated cells. In particular, the inhibition, or ‘knocking out’, of cathepsin L led to increased radiosensitivity, as documented by increases in cell death [33]. Their study highlights the possibility of targeting cathepsin L as a therapeutic strategy to enhance the effectiveness of radiotherapy.

This Special Issue comprises a mixture of original papers and review articles, providing new insights into novel pharmacological entities that are already present or are facing the therapeutic landscape for the management of IBDs. In particular, special attention has been paid to the examination of new biomarkers, as well as the impact of gut microbiota manipulation and natural drugs in counteracting the chronic intestinal inflammation and visceral pain associated with IBDs. Finally, based on the growing incidence of CRC, a potential innovative therapeutic strategy able to inhibit the DNA repair mechanisms to enhance the effectiveness of radiotherapy has also been included. In conclusion, if successful, these approaches will significantly improve not only the management of IBDs but also the quality of life of individuals affected by these disorders. Finally, we would like to thank all authors for their outstanding contributions, and hope that this Special Issue will inspire further research and innovation in the pharmacological management of IBDs.

## Data Availability

Data is contained within the article.

## Abbreviations

ALCAR, acetyl-L-carnitine; CD, Crohn’s disease; CRC, colorectal cancer; DNBS, 2,4-dinitrobenzene sulfonic acid; FMT, fecal microbiota transplantation; IBDs, inflammatory bowel diseases; IEB, intestinal epithelial barrier; incRNAs, long non-coding RNAs; UC, ulcerative colitis

## References

[1] Muzammil M.A., Fariha F., Patel T., Sohail R., Kumar M., Khan E., Khanam B., Kumar S., Khatri M., Varrassi G. (2023). Advancements in Inflammatory Bowel Disease: A Narrative Review of Diagnostics, Management, Epidemiology, Prevalence, Patient Outcomes, Quality of Life, and Clinical Presentation. Cureus.

[2] Jayasooriya N., Baillie S., Blackwell J., Bottle A., Petersen I., Creese H., Saxena S., Pollok R.C. (2023). Systematic review with meta-analysis: Time to diagnosis and the impact of delayed diagnosis on clinical outcomes in inflammatory bowel disease. Aliment. Pharmacol. Ther..

[3] Selvakumar B., Samsudin R. (2025). Intestinal Barrier Dysfunction in Inflammatory Bowel Disease: Pathophysiology to Precision Therapeutics. Inflamm. Bowel Dis..

[4] Calvez V., Puca P., Di Vincenzo F., Del Gaudio A., Bartocci B., Murgiano M., Iaccarino J., Parand E., Napolitano D., Pugliese D. (2025). Novel Insights into the Pathogenesis of Inflammatory Bowel Diseases. Biomedicines.

[5] Hegarty L.M., Jones G.-R., Bain C.C. (2023). Macrophages in intestinal homeostasis and inflammatory bowel disease. Nat. Rev. Gastroenterol. Hepatol..

[6] Neurath M.F., Artis D., Becker C. (2025). The intestinal barrier: A pivotal role in health, inflammation, and cancer. lancet. Gastroenterol. Hepatol..

[7] Zhu M., Song Y., Xu Y., Xu H. (2023). Manipulating Microbiota in Inflammatory Bowel Disease Treatment: Clinical and Natural Product Interventions Explored. Int. J. Mol. Sci..

[8] Huang Y., Wu M., Xiao H., Liu H., Yang G. (2022). Mesalamine-Mediated Amelioration of Experimental Colitis in Piglets Involves Gut Microbiota Modulation and Intestinal Immune Cell Infiltration. Front. Immunol..

[9] Dahl J.-U., Gray M.J., Bazopoulou D., Beaufay F., Lempart J., Koenigsknecht M.J., Wang Y., Baker J.R., Hasler W.L., Young V.B. (2017). The anti-inflammatory drug mesalamine targets bacterial polyphosphate accumulation. Nat. Microbiol..

[10] Imbrizi M., Magro F., Coy C.S.R. (2023). Pharmacological Therapy in Inflammatory Bowel Diseases: A Narrative Review of the Past 90 Years. Pharmaceuticals.

[11] Chiba M., Tsuji T., Nakane K., Tsuda S., Ohno H., Sugawara K., Komatsu M., Tozawa H. (2022). Relapse-Free Course in Nearly Half of Crohn’s Disease Patients with Infliximab and Plant-Based Diet as First-Line Therapy: A Single-Group Trial. Perm. J..

[12] D’Amico F., Fasulo E., Jairath V., Paridaens K., Peyrin-Biroulet L., Danese S. (2024). Management and treatment optimization of patients with mild to moderate ulcerative colitis. Expert Rev. Clin. Immunol..

[13] Singh S., Velayos F.S., Rubin D.T. (2024). Common Instances of Low-value Care in Inflammatory Bowel Diseases. Clin. Gastroenterol. Hepatol. Off. Clin. Pract. J. Am. Gastroenterol. Assoc..

[14] Jiang F., Wu M., Li R. (2023). The significance of long non-coding RNAs in the pathogenesis, diagnosis and treatment of inflammatory bowel disease. Precis. Clin. Med..

[15] Chen G., Deng H., Li M., Fang X., He C., Shu Y., Wang F. (2024). The role of long non-coding RNA in Crohn’s disease. Heliyon.

[16] Baldan-Martin M., Rubín de Célix C., Orejudo M., Ortega Moreno L., Fernández-Tomé S., Soleto I., Ramirez C., Arroyo R., Fernández P., Santander C. (2023). Long Non-Coding RNA Signatures in the Ileum and Colon of Crohn’s Disease Patients and Effect of Anti-TNF-α Treatment on Their Modulation. Int. J. Mol. Sci..

[17] Subudhi R.N., Poonia N., Singh D., Arora V. (2024). Natural approaches for the management of ulcerative colitis: Evidence of preclinical and clinical investigations. Nat. Products Bioprospect..

[18] Minato I., Mena P., Ricciardiello L., Scaioli E., Belluzzi A., Rotondo E., Derlindati E., Montanini B., Michelini C., Tosi N. (2025). Evidence for a Modulatory Effect of a 12-Week Pomegranate Juice Intervention on the Transcriptional Response in Inflammatory Bowel Disease Patients Reducing Fecal Calprotectin Levels: Findings From a Proof-of-Principle Study. Mol. Nutr. Food Res..

[19] Li H., Ruan J., Huang J., Yang D., Yu H., Wu Y., Zhang Y., Wang T. (2023). Pomegranate (Punica granatum L.) and Its Rich Ellagitannins as Potential Inhibitors in Ulcerative Colitis. Int. J. Mol. Sci..

[20] Hurtado-Lorenzo A., Honig G., Weaver S.A., Larkin P.B., Heller C. (2021). Chronic Abdominal Pain in IBD Research Initiative: Unraveling Biological Mechanisms and Patient Heterogeneity to Personalize Treatment and Improve Clinical Outcomes. Crohn’s Colitis 360.

[21] Takahashi K., Khwaja I.G., Schreyer J.R., Bulmer D., Peiris M., Terai S., Aziz Q. (2021). Post-inflammatory Abdominal Pain in Patients with Inflammatory Bowel Disease During Remission: A Comprehensive Review. Crohn’s Colitis 360.

[22] Coates M.D., Clarke K., Williams E., Jeganathan N., Yadav S., Giampetro D., Gordin V., Smith S., Vrana K., Bobb A. (2023). Abdominal Pain in Inflammatory Bowel Disease: An Evidence-Based, Multidisciplinary Review. Crohn’s Colitis 360.

[23] Ferreira G.C., McKenna M.C. (2017). L-Carnitine and Acetyl-L-carnitine Roles and Neuroprotection in Developing Brain. Neurochem. Res..

[24] Chiechio S., Copani A., Gereau R.W., Nicoletti F. (2007). Acetyl-L-carnitine in neuropathic pain: Experimental data. CNS Drugs.

[25] Lucarini E., Micheli L., Toti A., Ciampi C., Margiotta F., Di Cesare Mannelli L., Ghelardini C. (2023). Anti-Hyperalgesic Efficacy of Acetyl L-Carnitine (ALCAR) Against Visceral Pain Induced by Colitis: Involvement of Glia in the Enteric and Central Nervous System. Int. J. Mol. Sci..

[26] Singh N., Baby D., Rajguru J.P., Patil P.B., Thakkannavar S.S., Pujari V.B. (2019). Inflammation and cancer. Ann. Afr. Med..

[27] Tripathi S., Sharma Y., Kumar D. (2025). Unveiling the link between chronic inflammation and cancer. Metab. open.

[28] Keller D.S., Windsor A., Cohen R., Chand M. (2019). Colorectal cancer in inflammatory bowel disease: Review of the evidence. Tech. Coloproctol..

[29] Biancone L., Armuzzi A., Scribano M.L., Castiglione F., D’Incà R., Orlando A., Papi C., Daperno M., Vecchi M., Riegler G. (2020). Cancer Risk in Inflammatory Bowel Disease: A 6-Year Prospective Multicenter Nested Case-Control IG-IBD Study. Inflamm. Bowel Dis..

[30] Wu S., Xie S., Yuan C., Yang Z., Liu S., Zhang Q., Sun F., Wu J., Zhan S., Zhu S. (2023). Inflammatory Bowel Disease and Long-term Risk of Cancer: A Prospective Cohort Study Among Half a Million Adults in UK Biobank. Inflamm. Bowel Dis..

[31] Oh J.M., Kim S., Tsung C., Kent E., Jain A., Ruff S.M., Zhang H. (2025). Comprehensive review of the resistance mechanisms of colorectal cancer classified by therapy type. Front. Immunol..

[32] Cervantes A., Adam R., Roselló S., Arnold D., Normanno N., Taïeb J., Seligmann J., De Baere T., Osterlund P., Yoshino T. (2023). Metastatic colorectal cancer: ESMO Clinical Practice Guideline for diagnosis, treatment and follow-up. Ann. Oncol. Off. J. Eur. Soc. Med. Oncol..

[33] Abdelaziz R.F., Hussein A.M., Kotob M.H., Weiss C., Chelminski K., Stojanovic T., Studenik C.R., Aufy M. (2023). Enhancement of Radiation Sensitivity by Cathepsin L Suppression in Colon Carcinoma Cells. Int. J. Mol. Sci..

